# Supplementary material for: Assessment of Pre-Clinical Liver Models Based on Their Ability to Predict the Liver-Tropism of Adeno-Associated Virus Vectors
Source: Hum Gene Ther. 2023 Apr 17;34(7-8):273–88. doi: 10.1089/hum.2022.188 (PMC10150726; doi:10.1089/hum.2022.188)
Supplement: Supplemental data [file Supp_TableS1.pdf]

Supplemental information

Supplementary Table 1. Components of human liver organoid expansion media

**Components**	**Concentration**	**Supplier**
Basal media:<br>Advanced DMEM/F-12 media<br>Penicillin-Streptomycin<br>HEPES<br>GlutaMAX	86%<br>Neat<br>1x<br>10 mM<br>1x	 Life Technologies 12634-028<br>Life Technologies 15140-122<br>Life Technologies 15630-056<br>Life Technologies 35050-061
Rspo1-conditioned media	10%	In-house. See reference 71
B-27 Supplement, without vitamin A	1x	Life Technologies 12587-010
N-2 Supplement	1x	Life Technologies 17502-048
Nicotinamide	10 mM	Sigma N0636
Human EGF	50 ng/ml	Peprotech AF-100-15
[Leu^{15}]-Gastrin I human	10 nM	Sigma G9145
N-acetylcysteine	1 mM	Sigma A0737
Human FGF-10	100 ng/ml	Peprotech 100-26
Human HGF	25 ng/ml	Peprotech 100-39
Forskolin	10 µM	Tocris Bioscience 1099
A83-01	5 µM	Tocris Bioscience 2939
