# Supplementary material for: Assessment of Pre-Clinical Liver Models Based on Their Ability to Predict the Liver-Tropism of Adeno-Associated Virus Vectors
Source: Hum Gene Ther. 2023 Apr 17;34(7-8):273–88. doi: 10.1089/hum.2022.188 (PMC10150726; doi:10.1089/hum.2022.188)
Supplement: Supplemental data [file Supp_TableS2.pdf]

Supplementary Table 2. Primers used in the presented study

Name	Sequence (5'->3')	
**ddPCR**	**Sequence (5'->3')**	
eGFP_F	TCAAGATCCGCCACAACATC	
eGFP_R	TTCTCGTTGGGGTCTTTGCT	
NHP_alb_F	CGCAACTCTTCGTGAAACCTATGG	
NHP_alb_R	CACATCAACCTCTGGTCTCACC	
NHP_b-actin_F (RT)	CAACGAGCGGTTCCGCTG	
NHP_b-actin_R (RT)	CAGCACTGTGTTGGCGTACAG	
**cDNA synthesis**	**Sequence (5'->3')**	
WPRE_R	GGATTTATACAAGGAGGAGAAAATGAAAG	
**Next-generation sequencing**	**Primer barcode (5'->3')**	**Main oligo sequence (5'->3')**
GFP_BC_WPRE_F01	GTTCA	GCTGGAGTTCGTGACCGCCG
GFP_BC_WPRE_F02	GTCAT	GCTGGAGTTCGTGACCGCCG
GFP_BC_WPRE_F03	CTGTA	GCTGGAGTTCGTGACCGCCG
GFP_BC_WPRE_F04	GTATT	GCTGGAGTTCGTGACCGCCG
GFP_BC_WPRE_F05	CTAGT	GCTGGAGTTCGTGACCGCCG
GFP_BC_WPRE_F06	ACTTC	GCTGGAGTTCGTGACCGCCG
GFP_BC_WPRE_F07	CCTAT	GCTGGAGTTCGTGACCGCCG
GFP_BC_WPRE_F08	ACTGA	GCTGGAGTTCGTGACCGCCG
GFP_BC_WPRE_F09	TCCAA	GCTGGAGTTCGTGACCGCCG
GFP_BC_WPRE_F10	GCATT	GCTGGAGTTCGTGACCGCCG
GFP_BC_WPRE_F11	TCAAG	GCTGGAGTTCGTGACCGCCG
GFP_BC_WPRE_F12	TCAGA	GCTGGAGTTCGTGACCGCCG
GFP_BC_WPRE_F13	TCGTA	GCTGGAGTTCGTGACCGCCG
GFP_BC_WPRE_F14	ACGAT	GCTGGAGTTCGTGACCGCCG
GFP_BC_WPRE_F15	TATCC	GCTGGAGTTCGTGACCGCCG
GFP_BC_WPRE_F16	CATGA	GCTGGAGTTCGTGACCGCCG
GFP_BC_WPRE_F17	CACTC	GCTGGAGTTCGTGACCGCCG
GFP_BC_WPRE_F18	GACTA	GCTGGAGTTCGTGACCGCCG
GFP_BC_WPRE_F19	TACCC	GCTGGAGTTCGTGACCGCCG
GFP_BC_WPRE_F20	GACAT	GCTGGAGTTCGTGACCGCCG
GFP_BC_WPRE_F21	GAAGA	GCTGGAGTTCGTGACCGCCG
GFP_BC_WPRE_R	CAACATAGTTAAGAATACCAGTCAATCTTTCAC	
