# Supplementary material for: Assessment of Pre-Clinical Liver Models Based on Their Ability to Predict the Liver-Tropism of Adeno-Associated Virus Vectors
Source: Hum Gene Ther. 2023 Apr 17;34(7-8):273–88. doi: 10.1089/hum.2022.188 (PMC10150726; doi:10.1089/hum.2022.188)
Supplement: Supplemental data [file Supp_FigS1.pdf]

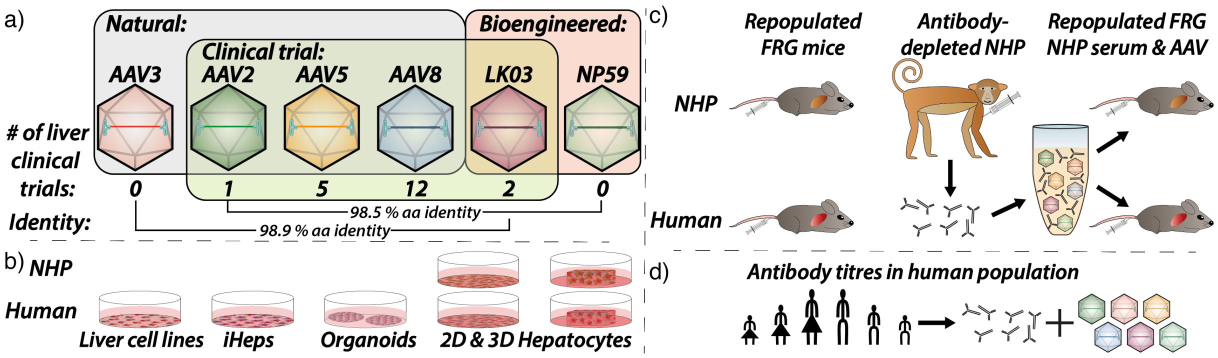

Supplementary Figure 1. Overview of the hepato-tropic AAV and pre-clinical liver model comparison. (**a**) AAVs chosen for this study based on their use in clinical trials or expected high performance in human hepatocytes based on previous studies and their amino acid identity. (**b**) Human and simian *in vitro* and *ex vivo* models of hepatocytes. (**c**) Human and simian *in vivo* models of hepatocytes. (**d**) Evaluation of antibody titres in human population for all six AAV variants included in the study. Abbreviations: AAV: adeno-associated virus; aa: amino acid; FRG: *Fah-/-/Rag2-/-/Il2rg-/-*; NHP: non-human primate
