# Supplementary material for: Assessment of Pre-Clinical Liver Models Based on Their Ability to Predict the Liver-Tropism of Adeno-Associated Virus Vectors
Source: Hum Gene Ther. 2023 Apr 17;34(7-8):273–88. doi: 10.1089/hum.2022.188 (PMC10150726; doi:10.1089/hum.2022.188)
Supplement: Supplemental data [file Supp_FigS2.pdf]

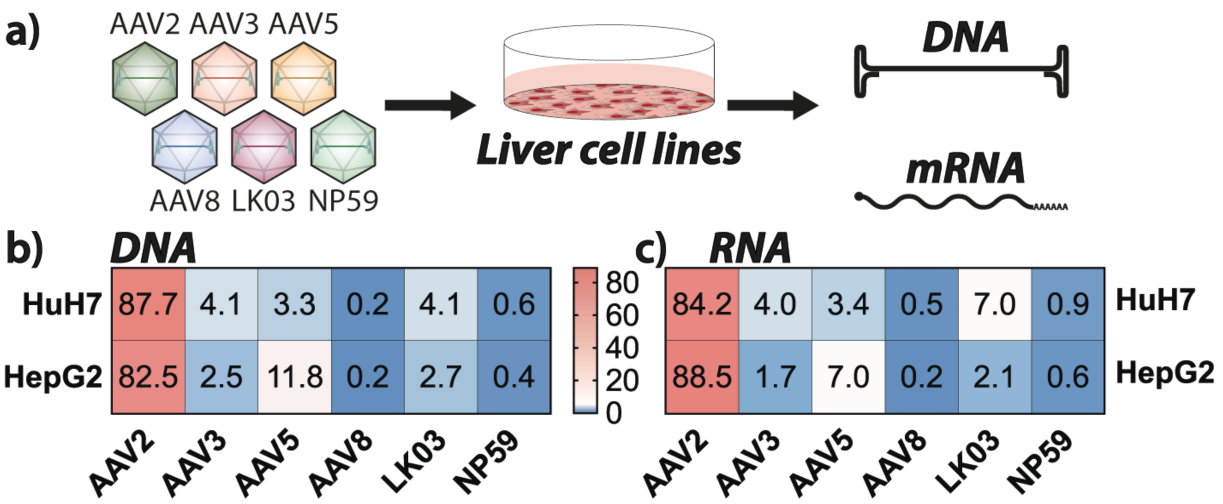

Supplementary Figure 2. AAV performance in hepatocellular carcinoma cell lines. (**a**) Schematic of transduction of indicated hepatocellular carcinoma cell lines. (**b**) NGS read contribution (%) for each AAV from extracted DNA. (**c**) NGS read contribution (%) for each AAV from mRNA-derived complementary DNA.
