# Supplementary material for: Assessment of Pre-Clinical Liver Models Based on Their Ability to Predict the Liver-Tropism of Adeno-Associated Virus Vectors
Source: Hum Gene Ther. 2023 Apr 17;34(7-8):273–88. doi: 10.1089/hum.2022.188 (PMC10150726; doi:10.1089/hum.2022.188)
Supplement: Supplemental data [file Supp_FigS3.pdf]

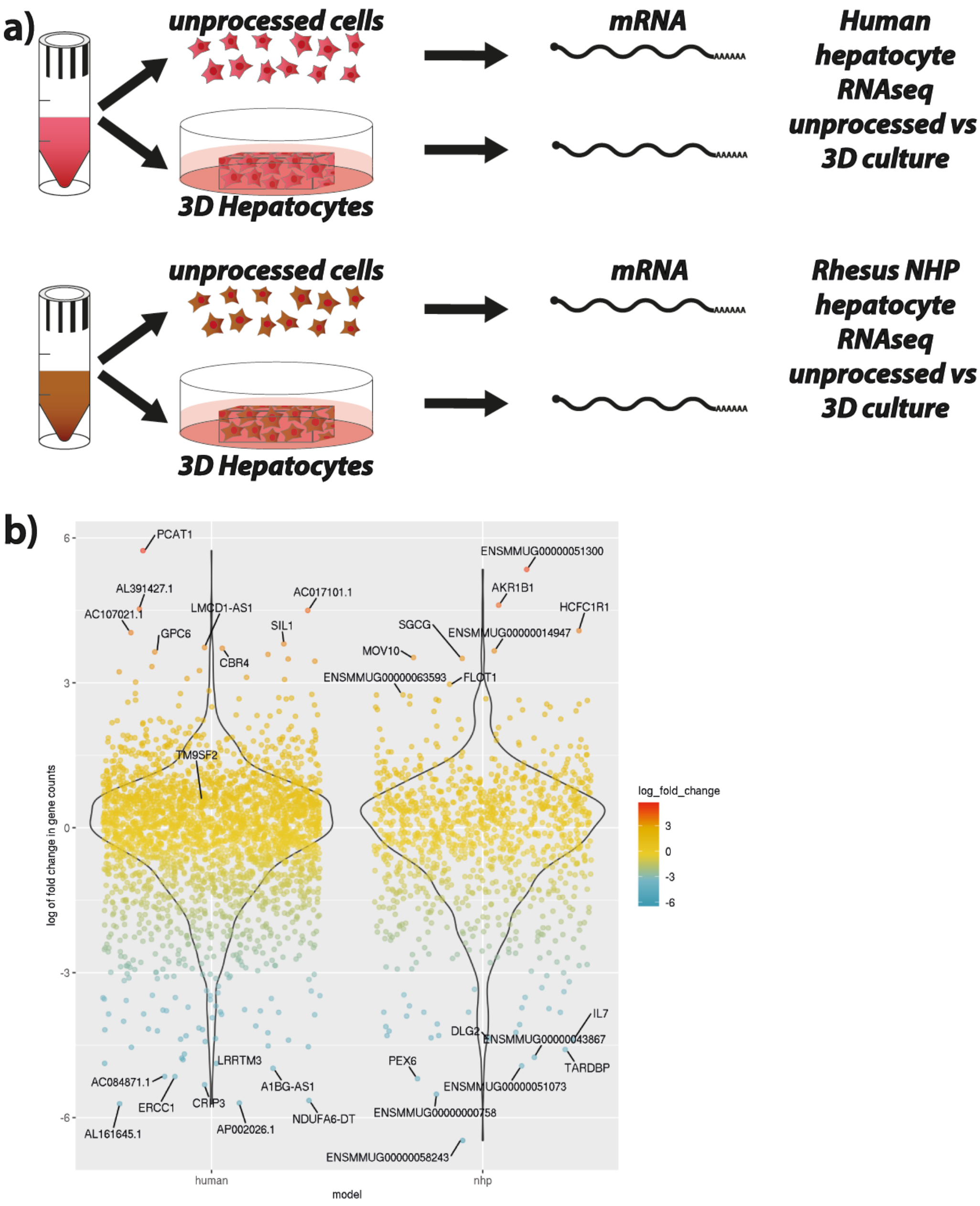

Supplementary Figure 3. Transcriptomic differences in 2D and 3D-cultured hepatocytes. (**a**) Schematic representation of experimental workflow. (**b**) Change in gene expression between normal and 3D cultured hepatocytes displayed as log of fold change.
