# Supplementary material for: Assessment of Pre-Clinical Liver Models Based on Their Ability to Predict the Liver-Tropism of Adeno-Associated Virus Vectors
Source: Hum Gene Ther. 2023 Apr 17;34(7-8):273–88. doi: 10.1089/hum.2022.188 (PMC10150726; doi:10.1089/hum.2022.188)
Supplement: Supplemental data [file Supp_TableS3.pdf]

Supplementary Table 3. Top 20 up- or down-regulated genes in 3D-cultured hepatocytes in printed hydrogels

gene	name	up/ down	gene type	function	antisense to	antisense function	TMM fold change
**ENSG00000253438.4**	**PCAT1**	**up**	**lncRNA**	**Inhibiting p53/ upregulating myc**	**N/A**	**N/A**	**53.37158**
ENSG00000224251.6	AL391427.1	up	lncRNA	unknown	AKR1C2	Aldo-keto Reductase	23.10789
ENSG00000227227.1	AC017101.1	up	lncRNA	unknown	ITGAV	Integrin sub-unit	22.62667
ENSG00000243415.2	AC107021.1	up	lncRNA	unknown	PLOD2	Collagen synthesis	16.44496
ENSG00000120725.13	SIL1	up	protein_coding	Nucleotide exchange factor	N/A	N/A	14.0066
ENSG00000227110.7	LMCD1-AS1	up	lncRNA	Antisense RNA	LMCD1	Zinc-finger, transcription cofactor	13.30003
ENSG00000145439.12	CBR4	up	protein_coding	Fatty acid biosynthesis	N/A	N/A	13.15157
**ENSG00000183098.11**	**GPC6**	**up**	**protein_coding**	**Heparan sulfate proteoglycan**	**N/A**	**N/A**	**12.47776**
ENSG00000225205.5	AC078883.1	up	lncRNA	unknown	ITGA6	Integrin sub-unit	12.03583
ENSG00000254733.1	AP001831.1	up	lncRNA	unknown	ME3	Malate dehydrogenase	11.27253
ENSG00000278518.1	AL161645.1	down	lncRNA	unknown	CYP2E1	Monooxygenase	0.01906202
ENSG00000246090.7	AP002026.1	down	lncRNA	unknown	ADH	Alcohol dehydrogenase	0.01927675
ENSG00000237037.9	NDUFA6-DT	down	lncRNA	unknown	NDUFA6	Mitochondrial membrane respiratory chain	0.01997939
ENSG00000146215.13	CRIP3	down	protein_coding	inflammation/cancer	N/A	N/A	0.0250615
ENSG00000012061.15	ERCC1	down	protein_coding	Nucleotide excision repair	N/A	N/A	0.02817534
ENSG00000251139.2	AC084871.1	down	lncRNA	unknown	ACSL1	Fatty acid biosynthesis	0.02820471
ENSG00000268895.6	A1BG-AS1	down	lncRNA	Antisense RNA	A1BG	Plasma glycoprotein	0.03172232
ENSG00000198739.11	LRRTM3	down	protein_coding	Synaptic acivity	N/A	N/A	0.03399672
ENSG00000231424.3	BX284613.2	down	lncRNA	unknown	FMO1	Monooxygenase	0.03403329
ENSG00000255240.6	AP001636.3	down	lncRNA	unknown	GLYATL1	Acyltransferase	0.03589633
