# Supplementary material for: Assessment of Pre-Clinical Liver Models Based on Their Ability to Predict the Liver-Tropism of Adeno-Associated Virus Vectors
Source: Hum Gene Ther. 2023 Apr 17;34(7-8):273–88. doi: 10.1089/hum.2022.188 (PMC10150726; doi:10.1089/hum.2022.188)
Supplement: Supplemental data [file Supp_FigS4.pdf]

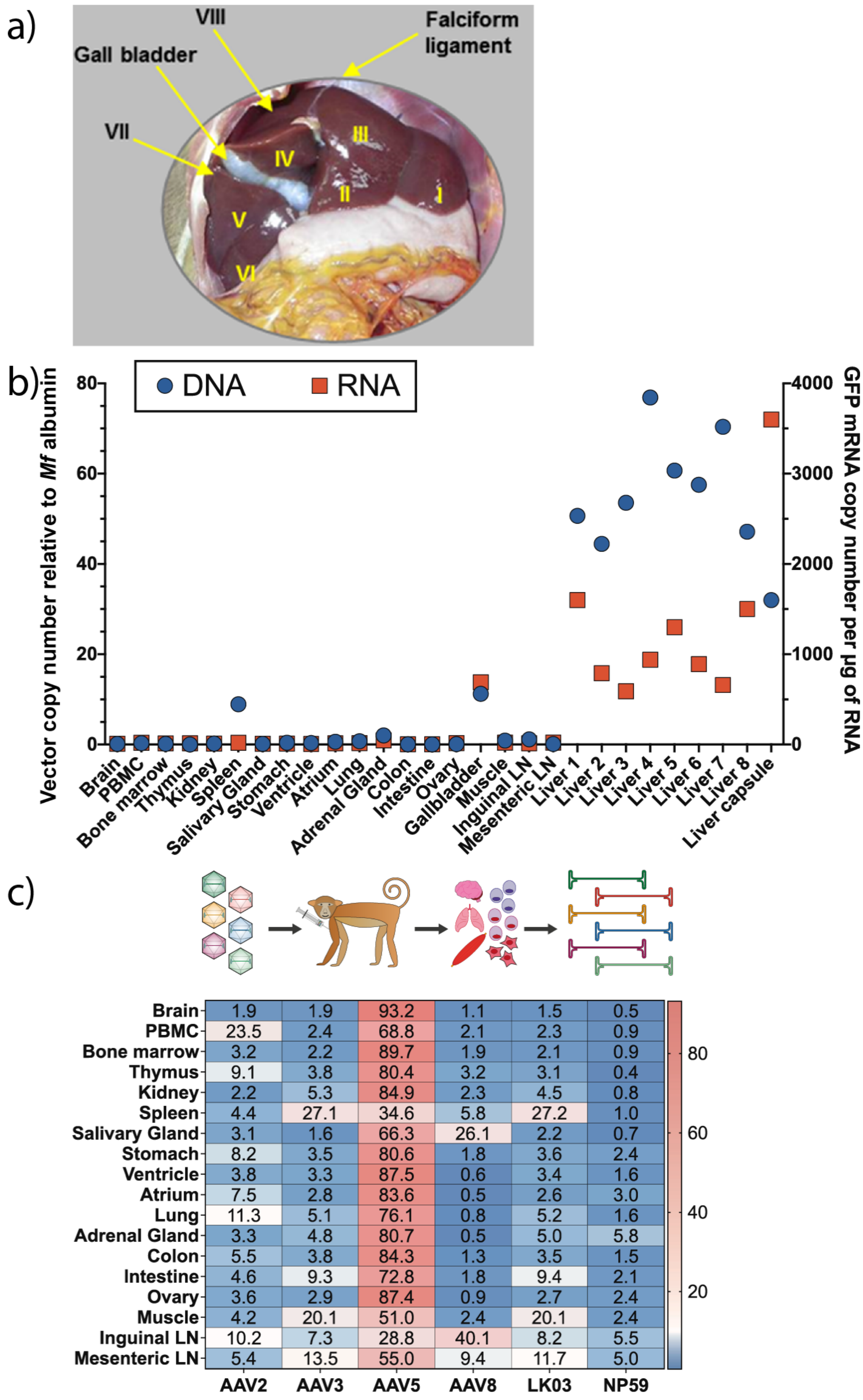

Supplementary Figure 4. Cynomolgus monkey AAV biodistribution. (**a**) Representation of liver regions indicated in **Figure 3**. (**b**) DNA (left y-axis) and mRNA/cDNA (right y-axis) vector copy number in the indicated organs and liver regions. (**c**) NGS read contribution (%) for each AAV from extracted DNA in the indicated cells and organs. Abbreviations: PBMC: peripheral blood-derived mono-nuclear cells; LN: lymph node
